# Community Pharmacists’ Perspectives on Antibiotic Misuse and Antimicrobial Resistance in Cyprus: A Reflexive Thematic Analysis

**DOI:** 10.3390/antibiotics15010045

**Published:** 2026-01-02

**Authors:** Mark J. M. Sullman, Timo J. Lajunen

**Affiliations:** 1 Department of Social Sciences, School of Humanities and Social Sciences, University of Nicosia, Nicosia 1700, Cyprus; sullman.m@unic.ac.cy; 2 Department of Psychology, Norwegian University of Science and Technology, 7491 Trondheim, Norway; 3 Department of Psychology, University of Helsinki, 00014 Helsinki, Finland

**Keywords:** community pharmacists, antibiotics, antimicrobial stewardship, antimicrobial resistance, qualitative research, Cyprus, reflexive thematic analysis

## Abstract

**Background:** Antimicrobial resistance (AMR) is a major global health threat, and Cyprus reports one of the highest levels of community antibiotic consumption in the EU. Despite their central role in antibiotic access and counselling, the stewardship practices and perspectives of community pharmacists in this regulated setting are not well documented. **Methods:** We conducted semi-structured qualitative interviews with 20 community pharmacists to explore their perspectives on antibiotic use and AMR. **Results:** We analysed the data using reflexive thematic analysis, revealing five key themes: regulation and control of dispensing; pharmacist–patient interaction and misuse; antimicrobial stewardship and public education; safety and professional responsibility; and systemic barriers. Pharmacists reported strict adherence to prescription-only rules, and described regulation and e-prescribing as a practical ‘shield’ that legitimised refusals and redirected some misuse from overt non-prescription requests towards attempts to reuse, extend, or ‘top up’ prior prescriptions and household leftovers. They described managing frequent patient demands for antibiotics for self-limiting conditions and using brief counselling scripts, written aids, and symptomatic alternatives to promote appropriate use. Participants emphasised the risks of antibiotic-related harms, including AMR and other health consequences, while also highlighting workload, access constraints, and communication difficulties as barriers to effective counselling. Overall, the findings indicate that community pharmacists in Cyprus function as front-line antimicrobial stewards. **Conclusions:** These accounts position community pharmacists in Cyprus as front-line antimicrobial stewards. Policy should consider supporting this function by providing enhanced communication tools, improving access pathways for timely prescriber review (including outside routine hours), and strengthening links between community pharmacy and national AMR action plans.

## 1. Introduction

Antimicrobial resistance (AMR) represents one of the most pressing threats to global public health, compromising routine clinical care and contributing to increased morbidity, mortality, and healthcare costs [1,2]. Estimates indicate that bacterial AMR contributed to approximately 1.27 million deaths in 2019 and was associated with a broader burden of about 4.95 million deaths that year [1]. The primary driver of this crisis is the excessive and inappropriate use of antibiotics, which has prompted worldwide efforts to monitor usage and promote antimicrobial stewardship [2]. Although antibiotic use in livestock, food production, and other non-human sectors also contributes to selection pressures, the present study focuses on community (outpatient) human antibiotic use and the stewardship role of community pharmacies.

Antibiotic consumption patterns show significant geographical variation. Cyprus exemplifies a high-consumption setting, reporting one of the highest per capita community antibiotic use rates in the EU during 2021–2022, including a 38% increase in that period—a trend concurrent with high national AMR levels [3]. These figures underscore the need to examine the determinants of antibiotic use at the point of access to care.

Cyprus has undergone major health-system reforms since the implementation of the General Healthcare System (GeSY) in 2019. Within GeSY, prescribing and dispensing are supported by a national information technology infrastructure (e-prescribing), and patients can obtain prescribed medicines from contracted community pharmacies, generating a traceable dispensing record that supports audit and regulatory oversight [4]. In the Republic of Cyprus, antibiotics are classified as prescription-only medicines and are legally supplied through licensed pharmacies; they are not legally available through supermarkets, markets, or other general retail outlets. Prior EU evidence suggests that when antibiotics are obtained without a prescription, they are most often sourced from pharmacies rather than other retail outlets or markets [5]. This system context is important because it shapes both the practical feasibility of non-prescription antibiotic access and the interactional work pharmacists perform when responding to patient demand.

Community pharmacists occupy a central role in the pathway from prescription to patient use. Their responsibilities extend beyond dispensing to include patient counselling on adherence and dosing, and they often act as an accessible first port of call for minor ailments, sometimes functioning as de facto primary care providers [6,7]. This position carries significant responsibility, as dispensing without a prescription or providing inadequate counselling heightens the risk of inappropriate use and AMR [8,9]. International evidence confirms that interventions combining regulation, pharmacist training, and public education reduce non-prescription antibiotic supply and enhance stewardship [10,11].

The drivers of inappropriate antibiotic use are multifactorial. Structural barriers, including long waiting times for appointments, high consultation costs, geographic inaccessibility of services, and limited diagnostic capacity, encourage a pharmacy-first approach and self-medication [6]. These are compounded by behavioural and social determinants, such as persistent public misconceptions (e.g., believing antibiotics treat viral respiratory infections), equating severe symptoms with bacterial cause, and relying on advice from friends or family, all of which fuel patient demand [12,13]. The imperative for stewardship is further strengthened by emerging evidence linking antibiotic exposure to broader long-term health consequences, including gut microbiome disruption and associated chronic physical and mental health conditions [14,15,16,17].

Individual personality and socio-cultural factors can also shape the behaviours that determine antibiotic use. For example, lower levels of conscientiousness and agreeableness have been associated with reduced adherence to prescribed antibiotic regimens [18], while societal norms can normalise self-medication and treatment advice from non-professionals [19,20]. Consequently, interventions focusing solely on prescribers or regulations are likely inadequate unless they also address the micro-level social processes and patient beliefs that drive demand.

Qualitative research is particularly well suited to unravel these complex contextual drivers. Our recent study in Brazil employed reflexive thematic analysis of semi-structured interviews with community pharmacists to explore how access constraints, public beliefs, adherence behaviours, professional relationships, and uneven regulatory enforcement interact to sustain non-prescription antibiotic use [6]. Our study identified six interrelated themes—Access and Self-Medication; Relationships with Healthcare Professionals; Knowledge and Beliefs about Antibiotics; Use and Adherence; Healthcare System Barriers; and Regulation and Enforcement—that provide a framework for developing multi-faceted stewardship strategies [6].

Our Cypriot study goes beyond a simple replication by examining how a comparatively stringent, digitally supported prescription-only environment reshapes the “sites” of misuse and the interactional work pharmacists must perform at the counter. Specifically, we focus on (i) how regulation and e-prescribing can function as a practical “shield” that legitimises refusals; (ii) how misuse may shift from overt OTC sales toward attempts to reuse, stretch, or “top up” existing prescriptions and household leftovers; and (iii) how pharmacists manage the dual positioning of community pharmacy as both a professional care setting and a commercial service environment. These mechanisms provide a clearer conceptual contribution than a purely descriptive comparison of ‘weak vs. strong’ enforcement.

Given Cyprus’s high community antibiotic consumption and documented public knowledge gaps [3,13,21], applying a similar pharmacist-centred qualitative approach can yield important insights into the local drivers of antibiotic use and identify feasible interventions. Building on international evidence supporting pharmacy-based antimicrobial stewardship strategies, including standardised counselling scripts, pictogram-based (and where needed multilingual) dosing aids, and coordinated enforcement [10,22,23], this study aims to explore community pharmacists’ attitudes, knowledge, and behaviours regarding antibiotic use and stewardship in Cyprus, and to identify the legal, economic, and socio-cultural barriers and facilitators to optimal practice.

## 2. Results

The analysis of 20 Greek-transliterated pharmacist interviews produced five themes that recurred across all five reflexive thematic analysis runs. We report these themes as pharmacists’ observations about patient behaviour and lay understandings, with the aim of describing the practice setting rather than presenting patient testimony. Table 1 provides a synopsis of each theme, showing how many interviewees mentioned each, the total number of coded segments, and a short thematic label. Counts of interviewees and coded segments are presented for descriptive transparency and should not be interpreted as statistical estimates of prevalence. Themes related to regulation and control of antibiotic dispensing and to pharmacist–patient interaction/misuse were especially prominent in the dataset, suggesting that they were central in pharmacists’ accounts of dispensing decisions. Even the least common theme, Systemic and Practical Barriers, appeared in 12 interviews (35 references), indicating that it remained a relevant element of pharmacists’ accounts. Notably, pharmacists did not spontaneously describe point-of-care testing as an existing routine practice or as a salient part of their stewardship work. Accordingly, we treat point-of-care testing as a potential future adjunct discussed in relation to the wider literature and system needs, rather than as a theme grounded in these interview data.

### 2.1. Theme 1: Regulation and Control of Antibiotic Dispensing

Analysis revealed a strong, shared understanding that antibiotic supply is now embedded in a strict auditable prescription-only framework. Pharmacists described this system, which is supported by e-platforms, reimbursement rules, and inspections, as the main reference point in daily practice. Participants described the system as enabling them to redirect requests, explain refusals, and maintain uniform standards across pharmacies. The following extracts emphasise the strong role of rules and the system:

“Antibiotics are strictly prescription-only now; without a doctor’s order we don’t dispense.”

(Participant 4)

“If the prescription isn’t there, the software blocks the sale… it’s clear and simple now: no prescription, no medicine.”

(Participant 11)

“GESY has standardised the process—everything is recorded.”

“Enforcement gives us legitimacy when we tell patients ‘no’.”

(Participant 6)

These accounts show pharmacists positioning the law and the electronic system as an external authority—refusal is presented as obligatory rather than personal. Several interviewees also described routine contact with prescribers to clarify unusual quantities or expired prescriptions: “If the quantity looks odd, we call the doctor to confirm before dispensing” (Participant 15), indicating that regulation is accompanied by cooperative verification rather than isolated gatekeeping. Pharmacists reported several common control scenarios that illustrate how regulation is enacted in practice: (i) expired or incomplete scripts that trigger system warnings and require prescriber confirmation; (ii) repeat-supply requests without review, redirected to the prescriber before any dispensing decision; and (iii) cross-pharmacy consistency, where the same e-rules apply regardless of pharmacy, reducing the incentive to “shop around.” Overall, participants described control mechanisms as helping to protect pharmacists and patients by standardising antibiotic dispensing.

### 2.2. Theme 2: Pharmacist–Patient Interaction and Misuse

Across interviews, the medicines counter emerged as a site of active negotiation, even within a strict prescription-only regime. Pharmacists described patients arriving with firm expectations of receiving immediate antibiotic treatment, often grounded in prior self-treatment, family advice, or access to leftover medicines. Managing these encounters required simultaneous education and boundary-setting, translating system rules into brief, patient-centred explanations that could be delivered under time pressure.

“Some arrive convinced they need antibiotics because it ‘worked last time’.”

(Participant 7)

Threats to “go elsewhere” were reported as a recurrent tactic, which pharmacists addressed by stressing system-wide consistency rather than individual discretion.

“A few say they’ll go to another pharmacy that will give it—still, we refuse.”

(Participant 5)

The tempo and tone of interactions were frequently shaped by immediacy, with participants noting that urgency intensified expectations and narrowed attention.

“The hardest part is urgency: they want antibiotics today.”

(Participant 16)

To defuse pressure, pharmacists used a short sequence: recognise the worry; restate the prescription requirement; give a brief clinical reason against antibiotics for likely viral cases; offer over-the-counter symptom relief and clear safety-netting (return/urgent review criteria); and, where uncertainty remained, refer to the prescriber. The sequence was described as helping to preserve rapport and to avoid inappropriate or unsafe supply.

“We reframe expectations and suggest symptomatic care first. Once we explain calmly, most accept it; a minority get agitated.”

(Participant 8)

Participants reported three common misuse patterns that triggered brief counselling: use of leftover stock at symptom onset, unsupervised extensions of a completed course, and intra-household sharing. The main concern was continuation without review. In response, pharmacists declined supply, outlined risks from extended or intermittent dosing, and directed patients to the prescribing clinician for reassessment.

“People finish one course and then ask for another box on their own—it can go 20–30 days.”

(Participant 12)

The pharmacists encountered “pharmacy shopping” by emphasising uniform rules and electronic controls across pharmacies, which reframed refusal as adherence to shared standards rather than a personal judgment. In sum, interactional work makes regulation usable in real encounters: pharmacists turn abstract controls into brief, respectful explanations, set firm boundaries, and redirect requests toward safer, evidence-aligned care.

### 2.3. Theme 3: Antimicrobial Stewardship and Public Education

Across interviews, pharmacists framed day-to-day counselling as part of a long-running effort to curb unnecessary antibiotic use. They described stewardship as work done at the counter—short, repeatable messages during dispensing or refusal—reinforced by system features (standardised processes, audit feedback) and public campaigns. Participants said these consistent signals have made conversations smoother, with more patients arriving already aware that antibiotics are not the default.

“Campaigns make people think twice before requesting antibiotics.”

(Participant 9)

Pharmacists linked campaign visibility with a more receptive starting point for counselling and with fewer “automatic” requests for antibiotics in common, self-limiting conditions. Consistency and repetition across multiple channels such as media outcomes, practice posters, pharmacy leaflets, and the same message from different professionals, were seen as central to shifting expectations.

“Consistent messaging across channels works best—the public recognises that antibiotics aren’t for every cold.”

(Participant 10)

Pharmacists cast themselves as frontline stewards, turning system rules into concrete actions. They corrected viral–bacterial misunderstandings, checked whether previous courses were completed, discouraged using leftovers, and gave safety-net advice (what to watch for, when to seek review). Several said they use short counselling “scripts” and teach-back to check understanding, backed by printed dosing reminders or multilingual leaflets when needed. They also framed refusals as stewardship: clear boundaries, a brief rationale, and immediate symptomatic alternatives.

“We are the first point of contact—stewardship starts with us. Saying ‘no’ kindly is sometimes the most responsible act.”

(Participant 1)

Participants further perceived that standardised e-prescribing, dispensing checks, and periodic audits reduced variability between pharmacies, which they felt reinforced the credibility of the stewardship message. They perceived a tangible improvement over earlier practice environments, with fewer casual or convenience-driven prescriptions reaching the counter. In their accounts, electronic controls and audit trails did not replace counselling; participants described these features as amplifying counselling by making decisions traceable and expectations clearer for both patients and professionals.

“Overprescribing was worse in the past; with electronic systems and audits it’s much improved.”

(Participant 18)

In sum, pharmacists described stewardship as operating on several levels: public campaigns shape expectations; system standardisation keeps prescribing and dispensing thresholds consistent; and brief, counter-based counselling turns these signals into practical advice. Participants described education and regulation as mutually reinforcing—public messages primed acceptance, while the pharmacist’s explanation at the point of care anchored behaviour in immediate choices (finish the course, avoid leftovers, seek follow-up when needed).

### 2.4. Theme 4: Safety and Professional Responsibility

Across interviews, pharmacists framed final dispensing decisions as safety-led and grounded in professional conscience, not merely rule-driven. Safety was most pressing when clinical information was incomplete (e.g., missing history, unclear indication) or when potential harm (allergy, interactions, contraindications) could not be quickly ruled out at the counter. Participants presented this stance as both personal ethics and a shared professional norm that places patient protection above convenience or commercial interests.

“We’re cautious—an antibiotic allergy can be fatal; without the history we can’t take that risk.”

(Participant 2)

In practice, pharmacists described a structured approach when safety was uncertain: pause dispensing; clarify the indication and any prior reactions where possible; check for duplication or interactions (especially with recent or concurrent antibiotics); and—if doubt remained—refer to the prescriber rather than proceed on assumption. When records were incomplete or the patient could not provide key details, the default was to avoid irreversible decisions at the counter and to prioritise re-assessment.

“Safety overrides convenience every time.”

(Participant 14)

Participants emphasised that this stance extended beyond acute risks. They cited dose- and duration-related concerns (e.g., unusually high dose requests; requests to “top up” a completed course), population-specific caution (e.g., pregnancy, paediatrics, significant comorbidities), and stewardship issues (e.g., unnecessary broad-spectrum choices). In such situations, pharmacists used short, clear explanations to justify refusal or delay, paired with safety-net advice and documentation of the encounter. Several interviewees also described calling the prescriber to confirm the plan when something “did not add up,” viewing prescriber–pharmacist dialogue as part of their duty of care rather than a challenge to prescriber authority.

“It’s about professional conscience—not pushing sales. We refuse even if it costs a sale; that’s the right thing to do.”

(Participant 17)

The interviewees acknowledged the commercial setting of community pharmacy as a potential source of pressure, but participants were explicit that sales considerations do not determine antibiotic decisions. Instead, they highlighted boundary practices that protect clinical judgement: separating clinical checks from payment steps; using documented standard operating procedures for refusals; and referring to uniform system rules to reduce any perception of arbitrariness. These boundaries were described as helping to sustain trust with patients, even when the outcome was not what initially sought.

“Delaying is better than harming.”

(Participant 3)

Deliberate delay was used when essential information was missing, when red flags warranted medical review, or when the requested use was clearly unsafe (e.g., unsupervised continuation after a completed course). Pharmacists paired delay with clear, actionable alternatives (symptom-relief options, warning signs that require prompt care, and instructions to contact the prescriber), turning refusal into a safety intervention rather than a dead end.

Together, these accounts position pharmacists as clinical gatekeepers whose core obligation is framed as preventing harm. While structure is provided by rules and electronic controls, decisions depend on a consciously held ethic: In the case of uncertain information or considerable risk, caution, verification, and referral are essential. Patient safety and professional responsibility are not add-ons to regulation; they guide how regulation is enacted at the point of dispensing.

### 2.5. Theme 5: Systemic and Practical Barriers

Across interviews, pharmacists described how day-to-day operating conditions (workload peaks, after-hours access to prescribers, communication barriers, and occasional system frictions) constrained the depth of counselling and the ease of enforcing policy. Importantly, participants reported maintaining the prescription-only standard even when timely prescriber access, including out-of-hours, was limited; in these situations, they continued to refuse non-prescription antibiotic requests and instead offered appropriate over-the-counter symptomatic treatments for likely viral, self-limiting illness, alongside safety-net advice and signposting to prescribers or out-of-hours services. Despite these pressures, participants emphasised decisions that remained aligned with the prescription-only rule, relying on brief, structured conversations and clear safety-netting when time or access was limited.

“Busy hours limit how deep we can go in counselling—we prioritise the essential points.”

(Participant 19)

Time pressure prompted a triage style of counselling: pharmacists reported using concise, repeatable scripts that foregrounded (i) the prescription requirement, (ii) a short rationale for non-antibiotic care when appropriate, and (iii) specific symptom-relief options. When queues were long, key messages were reinforced with written labels or printed leaflets to reduce the risk of misunderstanding. Several interviewees noted that this “essential points” approach helped maintain consistency during peak periods without diluting core stewardship content.

“During weekends or night shifts, reaching a doctor for approval can be difficult.”

(Participant 13)

Limited prescriber availability outside routine hours was described as a recurrent barrier that increased pressure for immediate supply. In these situations, participants reported pausing dispensing decisions that required prescriber input (e.g., clarifying indication, dose, or duration) and substituting interim guidance. Where uncertainty persisted, pharmacists documented the encounter and directed patients to appropriate out-of-hours care or to the prescriber at the earliest opportunity, framing delay as a safety measure rather than a refusal without remedy.

“We provide symptomatic alternatives until a script is obtained, but we don’t bypass the process.”

(Participant 20)

A common bridging strategy was to offer OTC symptomatic medicines with clear instructions while preserving the integrity of the prescribing pathway. Participants highlighted the importance of explicit safety-net advice (what to monitor, when to seek reassessment) to ensure that temporary measures did not drift into unsupervised antibiotic use. This approach was also applied to requests to extend a completed antibiotic course, where pharmacists declined supply but provided practical relief options and directed patients back to clinical review.

“Policy consistency matters even when access is limited.”

(Participant 6)

Uniform messaging across pharmacies and stable electronic rules were seen as crucial to managing expectations during difficult access windows. Interviewees said that consistent policy reduced “pharmacy shopping” and made brief explanations more credible, especially when language or communication barriers were present. To support comprehension, pharmacists described using simplified instructions, visual aids, and (where available) multilingual materials, particularly for patients unfamiliar with local health-system procedures.

Taken together, these accounts depict antibiotic governance as work conducted in real service conditions: pharmacists balance throughput and patient needs with a safety-first, policy-consistent stance. When workload, timing, or communication issues limit full counselling, they compensate with shorter scripts, written reinforcements, and symptom-relief advice—while deferring antibiotic supply until a valid prescription and necessary clinical checks are secured. This operational repertoire may help maintain access to symptom relief and support patient safety without compromising the core standard for antibiotic dispensing.

## 3. Discussion

This qualitative study explored how community pharmacists in Cyprus understand and manage antibiotic use and antimicrobial resistance (AMR) within a relatively strict prescription-only system. Beyond confirming the importance of prescription-only regulation, the novel contribution is to examine how digitally enforced governance may change the form and site of misuse, and how pharmacists enact “boundary work” by using regulation as an interactional resource to refuse supply while maintaining care, rapport, and safety through symptomatic alternatives and referral. Using reflexive thematic analysis of interviews with pharmacists from different districts and pharmacy types, we identified five interconnected themes: regulation and control of antibiotic dispensing; pharmacist–patient interaction and misuse; antimicrobial stewardship and public education; safety and professional responsibility; and systemic and practical barriers. Collectively, these themes suggest that pharmacists act as everyday gatekeepers who translate national rules and professional norms into practical decisions at the point of dispensing. Their accounts contextualise antibiotic use within a setting of high community consumption and persistent public misconceptions in Cyprus [3,13,21], consistent with global evidence linking inappropriate antibiotic use to the growing AMR burden [1,2].

A key difference emerges from comparison with the foundational Brazilian study. In Brazil, pharmacists identified uneven enforcement and a pervasive culture of self-medication as central challenges [6]. In Cyprus, participants described a clear, shared adherence to a “no prescription, no antibiotic” rule, underpinned by electronic prescribing, pharmacy software, and clear legislation. This is consistent with international evidence that firm regulatory reforms can curtail over-the-counter sales [11,24,25,26]. However, pharmacists’ accounts also indicate that formal rules do not eliminate pressure from customers. Pharmacists reported persistent attempts by patients to obtain antibiotics without a prescription, reuse old prescriptions, or utilise leftover medicines, behaviours also documented in Brazil and other Latin American contexts [6,27,28]. Thus, while strong legal controls form a necessary foundation for stewardship, they are an incomplete solution, as pharmacists must continually explain and uphold these rules in routine encounters.

Conceptually, our findings can be interpreted as pharmacists’ “boundary work”. Pharmacists positioned themselves as accessible health professionals (offering assessment, advice, and symptomatic relief) while simultaneously enforcing professional and legal limits on antibiotic supply. Participants described regulation and e-prescribing as a practical “shield” that helped make refusals legitimate and less personal (for example, “the system/law does not allow this”), which they perceived as reducing conflict and supporting consistent practice across pharmacies. This helps specify a mechanism by which strict enforcement may matter: it does not remove patient demand, but it may change the interactional resources available to pharmacists when negotiating that demand.

Our theme on pharmacist–patient interaction illustrates the practical reality of these encounters. Pharmacists described an ongoing tension between being approachable and helpful, and refusing inappropriate antibiotic requests. Consistent with survey findings from Cyprus and elsewhere, they reported that many patients still perceive antibiotics as “strong” medicines or a quick remedy for common respiratory and other self-limiting conditions [8,12,13]. These patient expectations mirror lay logics observed in Brazil, where symptom severity and past experience often supersede medical indications in guiding requests [6]. In Cyprus, pharmacists described a form of boundary work: they declined non-prescription supply and resisted requests for “top-ups” and early repeats, while striving to preserve the professional relationship by explaining their clinical reasoning, suggesting symptomatic treatments, and providing clear guidance on when to consult a doctor. This dual role in triage and counselling under high demand and constrained medical access is also reflected in national surveys and simulated-patient studies from Brazilian pharmacies [29,30,31].

The theme of antimicrobial stewardship and public education demonstrates how pharmacists described putting this boundary work into practice. Participants reported using brief, repeatable scripts that combined a firm statement of the prescription rule with a concise clinical explanation (e.g., “this is probably viral,” “antibiotics won’t speed recovery”) and suggestions for over-the-counter symptom relief. Some reinforced these messages with simple written instructions or printed leaflets. These strategies are consistent with studies reporting that structured counselling and clear educational materials at the point of dispensing can improve adherence and reduce misuse [9,22]. They also align with commentaries positioning community pharmacists as stewards when education is embedded into routine practice rather than treated as a separate activity [7]. However, pharmacists noted that queues, time pressure, and language barriers, particularly with tourists and migrants, often forced them to abbreviate counselling. In these situations, they reported relying on a core set of messages, visual aids, and, when available, multilingual leaflets, approaches consistent with research on pictograms, low-literacy communication, and myth-busting via social-media [12,22].

When discussing safety and professional responsibility, pharmacists’ concerns extended beyond AMR. They frequently cited the risks of allergic reactions, gastrointestinal side effects, complications from inappropriate doses and durations, and concerns about repeated courses in vulnerable populations such as children and older adults. These accounts are consistent with a safety-first professional ethos that transcends mere rule-following. They sit alongside evidence linking repeated antibiotic exposure to gut microbiome disruption and potential long-term consequences for metabolic, immune, and mental health [14,15,17]. Mirroring our Brazilian findings, pharmacists in Cyprus often framed refusal or delay as a protective measure for both the individual and the community [6]. We interpret this ethical framing as a potential cornerstone for stewardship efforts designed to support pharmacists in questioning or refusing inappropriate antibiotic use.

Finally, the theme of systemic and practical barriers shows how the wider health system constrains and shapes what happens at the point of dispensing. Although the General Health System (GESY) provides relatively broad formal access to primary care, pharmacists described difficulties contacting prescribers outside office hours, appointment delays, and practical obstacles related to work, childcare, and transport. Participants described these system-level pressures as increasing expectations for an immediate pharmacy-based solution, including in clinically inappropriate situations. Prescriber-side factors may also contribute to inappropriate antibiotic use, including prescribing decisions (e.g., selection, duration, and repeat issuing) and the organisation of prescribing pathways, which may interact with patient expectations and access constraints. Importantly, pharmacists’ accounts indicate that refusal to supply antibiotics without a prescription was maintained even when out-of-hours prescriber access was constrained, with pharmacists mitigating this pressure through interim symptomatic management, clear safety-netting, and referral for timely medical review. In response, pharmacists reported using “bridging” strategies: recommending symptomatic treatments, providing clear safety-net advice, and insisting on medical review for warning signs, without relaxing prescription-only requirements. Structural analyses from Brazil and other high-use settings have reported similar patterns, where long waits, costs, and staff shortages drive pharmacy-first consultations and self-medication [11,27]. International work on point-of-care diagnostics suggests that tools such as C-reactive protein testing in community pharmacies, combined with training and defined referral pathways, can reduce inappropriate antibiotic supply in some settings [10,23]. While not yet routine in Cyprus, pharmacists’ reports of diagnostic uncertainty and recurrent “borderline” cases suggest that carefully designed pharmacy-based testing could support prescribers by informing decisions, rather than replacing clinical assessment.

Point-of-care testing (POCT) should therefore be interpreted as a cautious, system-level possibility rather than a primary data-grounded implication of this study. Our interview material did not generate POCT as a salient theme; we therefore discuss it only as a potential adjunct in selected conditions where evidence supports its use and where clear training, governance, quality assurance, and referral pathways exist [10,23].

In summary, community pharmacists in Cyprus are both regulated professionals and frontline observers of public behaviour. Like their Brazilian counterparts, they described a convergence of public misconceptions, family and peer advice, and system pressures at the pharmacy counter. In pharmacists’ accounts, the critical distinction lies in the manifestation of misuse: in Cyprus, strict prescription rules and electronic controls appeared to shift problematic behaviours towards attempts to bend or stretch existing prescriptions, rather than direct over-the-counter sales. This comparison suggests that stewardship strategies cannot be indiscriminately transplanted between countries. For Cyprus, interventions may be most effective if built on pharmacists’ existing regulatory “shield”, their routine brief counselling opportunities, and their awareness of the broader harms of antibiotics. Evidence that personality traits, family attitudes, and local narratives shape adherence and help-seeking [18] supports the rationale for combining pharmacy-focused measures with broader public campaigns and co-designed educational materials that directly address local beliefs and concerns [7,12,13].

### 3.1. Strengths and Limitations

This study has several notable strengths. The application of reflexive thematic analysis allowed for an in-depth exploration of how community pharmacists in Cyprus conceptualise antibiotic use, AMR, and their own professional responsibilities. By deliberately aligning our topic guide and analytic framework with our prior Brazilian study, we enabled a meaningful comparative analysis between two high-consumption countries with different regulatory environment [6]. Furthermore, the recruitment of pharmacists from more than one district and from both independent and chain pharmacies ensured that a spectrum of practice contexts was captured. Rigour was enhanced through comprehensive documentation of analytical decisions and regular team discussions, fostering transparency and reflexivity throughout the research process.

In contrast, there are several limitations that must be acknowledged. The use of purposive sampling and a sample of 20 pharmacists means that the findings are unlikely to be statistically representative, and we do not claim to have captured every potential perspective within Cypriot community pharmacies. As with all self-report data, the potential for social desirability and recall bias exists; participants may have underreported deviations from protocol or overemphasised idealised practices, particularly given the legal sensitivities surrounding antibiotic dispensing. Consistent with the principles of reflexive thematic analysis, the identified themes represent interpreted patterns of meaning and should not be construed as frequency-based measures of specific behaviours. The study’s scope was also limited to pharmacists’ viewpoints, omitting the valuable perspectives of patients, prescribers, or policymakers, and did not incorporate objective dispensing or prescribing data to triangulate the accounts. Lastly, the findings are intrinsically tied to the specific organisation and regulation of Cypriot community pharmacy and primary care, which may limit their direct transferability to jurisdictions with differing funding models, enforcement mechanisms, or professional roles. We did not cross-check pharmacists’ accounts with prescribing and dispensing data or with interviews from patients or prescribers (e.g., GP’s). Future research should combine these sources to better understand cause-and-effect relationships and figure out where in the process—prescribing, dispensing, or using medication—the most important changes can be made.

### 3.2. Future Directions

The results of this study highlight several productive avenues for future work. First, comparative qualitative or mixed-methods studies across European and Eastern Mediterranean countries with high community antibiotic consumption could help clarify how pharmacists’ stewardship roles are shaped by different regulatory, reimbursement, and primary-care arrangements. Direct comparisons between systems with stringent enforcement, such as Cyprus, and those with less consistent enforcement would be particularly valuable for understanding the interplay between legislation, commercial pressures, and public demand.

Second, there is a clear need for intervention studies to evaluate the practical strategies identified by our participants and supported by the broader literature. Promising approaches include standardised counselling prompts embedded into dispensing software, pictogram-based and multilingual aids for diverse patient groups, and improvements to care pathways that enable timely prescriber review (including after-hours). Feasibility testing of POCT should be pursued only cautiously and only where pharmacists, prescribers, and regulators jointly define indications, training, quality assurance, and referral criteria [9,10,22,23]. Future evaluations should assess not only changes in antibiotic dispensing rates but also secondary outcomes such as patient comprehension, satisfaction, re-consultation patterns, and equity of access.

Third, research that triangulates pharmacists’ accounts with other parts of the healthcare system is essential. Studies integrating the perspectives of pharmacists, prescribers, and patients, alongside routinely collected prescribing and dispensing data, could pinpoint critical decision points, identify root causes of misunderstandings, and determine which system levers—such as appointment availability, care continuity, or reimbursement rules—most effectively support or undermine appropriate antibiotic use [29,30,31,32].

### 3.3. Conclusions

This study demonstrates that community pharmacists in Cyprus are already integral to antimicrobial stewardship within a stringent prescription-only system. Conceptually, our findings indicate that stringent, auditable prescription-only systems do not in themselves eliminate demand for antibiotics; they reshape how misuse is attempted and provide pharmacists with a practical “shield” that supports boundary-setting and stewardship at the point of dispensing. They consistently uphold “no prescription, no antibiotic” regulations while navigating persistent patient demand driven by misconceptions, social networks, and systemic pressures. Their accounts reveal that refusing or delaying antibiotic supply is often framed as a protective intervention for patient and public health, moving beyond the concept of simple gatekeeping. Nonetheless, the effectiveness of this frontline stewardship is constrained by practical challenges, including time pressure, communication difficulties, and barriers to accessing timely primary care.

We contend that for antimicrobial stewardship efforts in Cyprus to be fully effective, policy should recognise community pharmacists as key partners in its design and implementation, rather than mere enforcers of rules. Empowering this role necessitates the continued consistent enforcement of prescription requirements, the provision of practical communication tools and targeted training, and systemic reforms that facilitate easier access to appropriate medical review. In countries with high community antibiotic consumption, more meaningful integration of community pharmacy into national AMR action plans will be essential for achieving and sustaining long-term improvements in antibiotic use. Because the present study is based on pharmacists’ self-report, system-level causal claims should be interpreted cautiously; future work should triangulate accounts with routine prescribing/dispensing data and with patient and prescriber perspectives.

## 4. Materials and Methods

### 4.1. Study Design

We conducted a qualitative study using semi-structured interviews with community pharmacists to investigate perceptions of antibiotic use, self-medication behaviours, and the drivers of antimicrobial resistance. The study followed the Consolidated Criteria for Reporting Qualitative Research (COREQ) to ensure comprehensive reporting for interviews and focus groups. Pharmacists’ accounts were treated as the primary unit of analysis; no patient-level or physician-level data were collected. To address COREQ reporting requirements without providing a standalone COREQ table, key participant, interview, and research-process characteristics are embedded in the Methods and reported in aggregate to protect anonymity.

### 4.2. Research Team and Reflexivity

Interviews were conducted by a trained member of the research team (see Acknowledgments), who had no supervisory relationship with participants. At the start of each interview, the interviewer explained the study purpose, voluntary participation, confidentiality, and the prescription-only legal context. Given the small-country setting, participant characteristics are reported in aggregate or in broad bands to support transparency while reducing re-identification risk.

### 4.3. Setting and Participants

The study was implemented across community pharmacies in the Republic of Cyprus, sampling urban and regional settings to capture variability in practice contexts. Eligible participants were registered community pharmacists working in retail settings, with at least one year of continuous professional practice, and willing to take part in an audio-recorded interview. Pharmacists employed solely in hospital pharmacies or exclusively in non-clinical administrative roles were excluded.

We recruited 20 pharmacists through purposive sampling aimed at achieving variation in geographic location, pharmacy type (independent and chain), and years of practice. Recruitment channels included professional associations, local pharmacy networks, and direct contact with pharmacy managers. In total, 20 pharmacists were approached; none declined participation, and no participants withdrew after initial contact.

Participant characteristics were collected to describe the sample and provide context for the analysis. However, due to the small size of the professional community, detailed demographic information is not presented to protect participant anonymity. Only summarized data on age, sex, and years since completing pharmacy studies are reported here.

Participants ranged in age from 24 to 65 years, with a median age of 38 years. The sample included 12 females and 8 males. The number of years since participants completed their pharmacy education ranged from 1 to over 40 years, with a median of approximately 13 years.

### 4.4. Data Collection

A semi-structured interview guide was developed following a targeted literature review and input from subject-matter experts. The guide addressed antibiotic dispensing practices, typical patient interactions at the counter, local barriers to appropriate antibiotic use, pharmacists’ understanding of antimicrobial resistance, and suggestions for interventions feasible in the Cyprus context. Interviews were offered in Greek or English according to participant preference. Interviews were conducted by a final-year clinical psychology student with prior research experience, who was trained and supervised by the first author to deliver the semi-structured interview guide in a consistent manner. Two initial interviews were conducted in October 2024; these were reviewed by both authors and judged to be of high quality, after which the same interviewer conducted the remaining interviews between December 2024 and June 2025. Interviews lasted approximately 45–90 min and were conducted one-to-one by the trained interviewer, under the supervision of experienced qualitative researchers; all interviews were audio-recorded with participants’ consent. Field notes were taken to capture contextual details and immediate analytic impressions. Participants were also asked about how patients typically access prescribers when antibiotics are requested, including out-of-hours situations; these accounts informed Theme 5 (Systemic and Practical Barriers).

### 4.5. Transcription and Data Management

Recordings were transcribed verbatim. Interviews conducted in Greek were translated to English for analysis. Transcription and, where applicable, machine-assisted translation were performed using a secure transcription platform, followed by manual verification and correction by a researcher fluent in the original language and English to preserve nuance and context. All transcripts were de-identified prior to analysis, with pseudonyms assigned and any potentially identifying details removed. Electronic data were stored on encrypted institutional servers in accordance with data protection guidelines.

### 4.6. Data Analysis

We used reflexive thematic analysis, based on Braun and Clarke’s approach [33,34], to identify patterns of meaning across pharmacists’ accounts. Analysis proceeded through the six canonical phases: (1) familiarisation with the data, (2) systematic coding, (3) searching for candidate themes, (4) iterative theme review and refinement, (5) defining and naming themes, and (6) preparing the analytic report. Coding was conducted in English on the final verified transcripts using qualitative analysis software to organise codes and extracts.

To assess the stability of interpretive claims, the analytic process was repeated across five independent iterative cycles, each time returning to the raw transcripts to re-examine code groupings and theme boundaries. This procedure supported analytic reflexivity and helped to confirm the coherence and resonance of the final theme set. Consistent with reflexive thematic practice, themes were understood as interpretive constructs developed by the research team rather than as statistical estimates of prevalence.

### 4.7. Trustworthiness and Reflexivity

Analytic decisions, codebooks, and theme memos were documented to provide an audit trail. Team discussions were convened regularly to surface assumptions, challenge interpretations, and resolve divergent readings of the data. Where translation occurred, the bilingual team member reviewed transcripts and translations to preserve local idiom and to reduce the risk of semantic loss. Findings are reported with illustrative quotations to convey participants’ perspectives while maintaining confidentiality.

### 4.8. Ethical Considerations

The study received institutional ethical approval from the Social Sciences Ethics Review Board of the University of Nicosia (SSERB 390; 7 August 2024). All participants provided written informed consent prior to participation. Confidentiality was preserved through de-identification of transcripts and secure data handling. Participants were informed of their right to withdraw at any time without consequence.

## Figures and Tables

**Table 1:** Emergent Themes and Sub-themes from Reflexive Thematic Analysis of 20 Interviews among pharmacists in Cyprus.

Theme	Subthemes	Mentions (All Interviews)	Interviewees (Out of 20)	Example Representative Quote
1. Regulation and Control of Antibiotic Dispensing	Prescription-only governance; collaboration with physicians; legal and policy enforcement	80	20	“Antibiotics are strictly prescription-only now; without a doctor’s order we don’t dispense.” (Participant 4)
2. Pharmacist–Patient Interaction and Misuse	Patient demand and insistence; self-medication and leftovers; counselling and adherence support	90	18	“Some arrive convinced they need antibiotics because it ‘worked last time’.” (Participant 7)
3. Antimicrobial Stewardship and Public Education	Awareness campaigns; decline in casual prescribing; professional responsibility	77	16	“Campaigns make people think twice before requesting antibiotics.” (Participant 9)
4. Safety and Professional Responsibility	Allergy and risk management; ethical decision-making	40	15	“We’re cautious—an antibiotic allergy can be fatal.” (Participant 2)
5. Systemic and Practical Barriers	Communication and language issues; time pressure; access challenges	35	12	“During weekends or night shifts, reaching a doctor for approval can be difficult.” (Participant 13)

## Data Availability

Data available on request due to privacy restrictions.
